# Tooth Extraction Timing After Head and Neck Radiotherapy: A Critical Reappraisal of Osteoradionecrosis Risk and Biological Vulnerability

**DOI:** 10.3390/mps9050149

**Published:** 2026-10-13

**Authors:** Erkan Topkan, Efsun Somay, Sibel Bascil, Ugur Selek

**Affiliations:** 1 Department of Radiation Oncology, Faculty of Medicine, Başkent University, 01250 Adana, Türkiye; docdretopkan@gmail.com or etopkan@baskent.edu.tr; 2 Department of Oral and Maxillofacial Surgery, Faculty of Dentistry, Başkent University, 06490 Ankara, Türkiye; 3 Department of Periodontology, Faculty of Dentistry, Başkent University, 06490 Ankara, Türkiye; bascil5@yahoo.com; 4 Department of Radiation Oncology, School of Medicine, Koç University, 34450 Istanbul, Türkiye; ugurselek@yahoo.com or uselek@ku.edu.tr

**Keywords:** tooth extraction, extraction timing, osteoradionecrosis of the jaws, radiation dose, biological vulnerability, selection bias

## Abstract

**Aims:** To critically reappraise the post-radiotherapy tooth-extraction waiting-period paradigm and examine whether extraction timing is better interpreted within a broader framework of biological vulnerability than as an isolated temporal determinant of osteoradionecrosis of the jaws (ORNJ) risk. **Methods:** This narrative review purposively synthesized clinical guidelines, systematic reviews, clinical and dosimetric studies, foundational radiobiological literature, and epidemiological methodology relevant to post-radiotherapy extraction timing and ORNJ. **Results:** Available evidence does not establish a universally safe post-radiotherapy extraction interval or demonstrate that progressively longer delays confer greater protection against ORNJ. Contemporary radiobiology supports persistent vascular, cellular, fibroatrophic, and bone-remodeling abnormalities after radiotherapy. Observational timing studies may also be susceptible to selection-related and time-dependent biases, although these mechanisms have not been demonstrated uniformly across the literature. Mandibular radiation exposure is consistently associated with ORNJ risk, whereas different dosimetric parameters are not interchangeable. Evidence for a dose–timing interaction currently derives from a single retrospective cohort and remains hypothesis-generating. **Conclusions:** Extraction timing should not be interpreted in isolation. Current evidence supports considering elapsed time together with mandibular radiation exposure, local tissue condition, extraction indication, surgical factors, and host-related determinants, without presuming that any fixed waiting interval is inherently protective.

## 1. Introduction

Post-radiotherapy tooth extraction constitutes a principal clinical precipitant of osteoradionecrosis of the jaws (ORNJ), a severe and functionally debilitating late complication of head and neck radiotherapy (RT). ORNJ is most often defined as exposed or necrotic irradiated bone that fails to heal for an extended period in the absence of persistent or recurrent malignancy [1,2]. Although advances in radiation delivery—particularly intensity-modulated radiotherapy (IMRT)—have reduced mandibular dose exposure in many contemporary settings, ORNJ continues to impose considerable morbidity, including chronic pain, infection, pathologic fracture, impaired oral function, and diminished quality of life [1,3,4,5]. As a result, determining the optimal timing for post-RT tooth extraction remains a critical and unresolved challenge in oral oncology and head and neck cancer survivorship care [6,7,8,9].

Delayed extraction is widely used as a precautionary strategy after RT, but systematic reviews have not established a universally protective post-treatment interval [8,9,10]. This uncertainty also has a biological dimension: classical and contemporary models of ORNJ describe persistent vascular, cellular, and fibroatrophic injury rather than a predictable return to baseline tissue function [11,12,13]. Whether elapsed time itself meaningfully improves the capacity of irradiated mandibular tissues to tolerate subsequent extraction therefore remains unresolved and represents a central question addressed in this review.

Interpretation of extraction-timing studies is further complicated by the non-random nature of clinical decision-making and by variation in mandibular radiation exposure, local tissue condition, and patient-specific factors [4,5,14,15,16]. This review therefore examines the historical origins of delayed-extraction recommendations, the contemporary biology of radiation injury, potential biases in observational timing studies, and the role of dosimetric and biological heterogeneity in modifying post-extraction ORNJ risk. The overarching question is whether extraction timing is best interpreted as an isolated temporal variable or within a broader framework of biological vulnerability. Here, biological vulnerability denotes the cumulative tissue-, treatment-, and host-related factors that influence ORNJ susceptibility following extraction in irradiated bone.

## 2. Review Scope and Literature Selection

This article presents a narrative review that critically examines the biological and methodological foundations of post-RT tooth extraction timing, rather than offering a systematic synthesis of the evidence. We purposively selected literature to enable a conceptual reappraisal of the waiting-period paradigm, emphasizing studies that elucidate the pathophysiology of osteoradionecrosis, post-RT dental extraction outcomes, mandibular dose–volume relationships, mechanisms of radiation-induced fibroatrophic injury, and methodological biases inherent in observational research. We prioritized clinical guidelines, systematic reviews, dose–volume analyses, contemporary cohort studies, and foundational radiobiological or epidemiological sources. Given the interpretive and hypothesis-generating purpose of this narrative review, we did not apply formal meta-analytic methods, structured risk-of-bias assessment, or PRISMA-guided study selection. Accordingly, the review should be interpreted as a critical synthesis of selected biological, clinical, dosimetric, and methodological evidence rather than as a systematic appraisal of the totality of the literature or as a source of pooled or quantitative estimates of the effect of extraction timing on ORNJ risk. Conclusions concerning the strength, consistency, or completeness of the evidence are therefore necessarily limited by the narrative review design.

## 3. Historical Origins of the Post-Radiotherapy Waiting-Period Paradigm

The practice of deferring tooth extraction following head and neck RT is well established in oral oncology. However, the rationale for a specific post-RT waiting interval is far less secure than its widespread application might imply. Contemporary systematic reviews consistently highlight the absence of universally accepted guidelines for the timing of dental surgery relative to RT, underscoring substantial heterogeneity in study design, extraction protocols, outcome definitions, and follow-up duration [7,8]. Consequently, the waiting-period paradigm is best regarded as a precautionary measure shaped by historical precedent rather than as a rigorously validated temporal standard.

This precautionary approach is rooted in early conceptual models of ORNJ. Marx’s influential 1983 framework reconceptualized ORNJ as a radiation-induced wound-healing disorder characterized by hypovascularity, hypocellularity, and tissue hypoxia, rather than as a primarily infectious process [11]. In this context, surgical trauma—especially tooth extraction—was viewed as a potential precipitant that could exceed the compromised reparative capacity of irradiated bone. Subsequent work by Epstein and colleagues expanded this perspective, describing ORNJ as a multifactorial entity resulting from the interplay of radiation injury, mucosal breakdown, local trauma, and secondary infection in susceptible tissues [12]. These foundational models provided a compelling biological rationale for minimizing elective dentoalveolar surgery after RT whenever feasible.

This biologically motivated caution progressively informed clinical practice. Pre-RT dental evaluation programs were established to identify and remove teeth with poor prognosis prior to treatment, thereby reducing the need for extractions in irradiated bone. Post-RT, clinicians have tended to adopt conservative dental management and delay extraction unless clinical circumstances—such as infection, pain, or non-restorability—necessitate intervention. This approach is understandable, given that post-RT tooth extraction has consistently been identified as a major risk factor for ORNJ in systematic reviews [6,7,8,9]. However, the evolution from general caution to specific waiting periods—such as several months, one year, or two years—appears to have been driven predominantly by institutional custom, retrospective observations, and expert opinion, rather than by robust prospective validation.

Multiple limitations inherent to the early evidence base complicate causal inference. Many foundational studies were conducted in pre-IMRT cohorts receiving two-dimensional or three-dimensional conformal RT, frequently involving high and poorly characterized mandibular dose exposures. These studies often lacked site-specific dosimetry, standardized ORNJ definitions, consistent extraction criteria, uniform surgical protocols, and detailed data on confounding variables such as smoking status, oral hygiene, periodontal health, chemotherapy administration, comorbidities, and dental follow-up [10,17]. Given these methodological constraints, associations between delayed extraction and reduced ORNJ incidence cannot be reliably ascribed to biological recovery over time. Alternative explanations—including differences in patient selection, extraction indication, radiation dose, or local tissue condition—are equally plausible.

The advent of IMRT has fundamentally altered the relevance of historical timing recommendations. IMRT provides superior dose conformity and enables greater sparing of uninvolved mandibular structures, contributing to reduced rates of mandibular ORNJ in contemporary cohorts [3,4]. Concurrently, contemporary dose–volume studies have reported strong associations of mandibular radiation exposure and irradiated bone volume with ORNJ risk [4,5,14,15]. These developments challenge the premise that elapsed time since RT should be the primary consideration in post-treatment extraction decisions. Mandibular sites receiving different levels of radiation exposure may therefore represent distinct biological environments for subsequent tooth extraction, irrespective of elapsed time since RT.

Recent systematic reviews reinforce this uncertainty. Beaumont et al. found no universally accepted optimal timing for dental surgery relative to RT [8], Lajolo et al. emphasized the limited and heterogeneous evidence concerning post-RT extraction-related ORNJ [7], and Corrao et al. advocated individualized risk assessment rather than reliance on a predetermined safe interval [9]. Collectively, these findings indicate that historical waiting-period recommendations should be regarded primarily as precautionary practice rather than evidence that elapsed time restores the healing capacity of irradiated tissues. This distinction frames the biological question addressed in the next section: how irradiated mandibular tissues evolve after RT.

Table 1 summarizes the major conceptual and evidentiary milestones that contributed to the emergence of the post-RT waiting-period paradigm.

## 4. Radiation Biology: Why Waiting May Not Restore Healing Capacity

The rationale for a post-RT waiting period rests on the expectation that irradiated tissues will recover or stabilize sufficiently over time to better withstand subsequent surgical intervention. Contemporary radiation biology, however, characterizes radiation-induced injury not as a transient insult followed by gradual normalization, but as a persistent, multifactorial process involving vascular compromise, cellular depletion, oxidative stress, fibrosis, and impaired tissue regeneration [11,13,18,19]. Accordingly, the assumption that delayed extraction inherently mitigates the risk of ORNJ lacks robust biological support.

The classical hypovascular–hypocellular–hypoxic model proposed by Marx provided the first comprehensive explanation for the impaired healing capacity of irradiated bone [11]. Under this paradigm, radiation exposure damages the microvasculature, reduces cellularity, and compromises oxygen delivery, limiting the ability of bone and surrounding soft tissues to respond adequately to injury. Within this framework, tooth extraction represents a traumatic event that may exceed the diminished reparative reserve of irradiated tissues, resulting in delayed healing, persistent bone exposure, infection, and eventual ORNJ. Although contemporary concepts have expanded upon Marx’s original model, the central principle remains highly relevant: irradiated jawbone possesses a substantially reduced capacity for wound repair.

Subsequent fibroatrophic theory has further refined the understanding of late radiation injury. Delanian and Lefaix described radiation as initiating a chronic cascade involving endothelial dysfunction, oxidative stress, dysregulated fibroblast activity, extracellular-matrix remodeling, vascular rarefaction, and progressive fibrosis [13]. Transforming growth factor beta-1 (TGF-β1) is considered a central mediator of radiation-induced fibroatrophy, promoting fibroblast activation, extracellular matrix deposition, and tissue fibrosis. Persistent dysregulation of these pathways may contribute to the chronic hypovascular, hypocellular, and poorly remodeling tissue environment characteristic of irradiated mandibular bone [13,19]. Rather than representing a stable state of tissue damage, irradiated tissues are subject to ongoing fibrovascular remodeling that can persist long after treatment has concluded. Contemporary studies of radiation-induced fibrosis corroborate this model, demonstrating that chronic fibrotic processes continually reshape tissue architecture, compromise vascular integrity, and diminish regenerative capacity over time [19]. This evolving paradigm directly challenges the notion that irradiated tissues progressively revert to their pre-treatment biological state.

These mechanisms have direct implications for extraction-socket healing. Successful healing after tooth extraction requires coordinated clot stabilization, inflammatory signaling, angiogenesis, fibroblast proliferation, granulation tissue formation, osteoid deposition, bone remodeling, and durable mucosal closure. Radiation exposure can disrupt each stage. Endothelial injury compromises neovascularization, fibroblast dysfunction impairs connective-tissue repair, chronic hypoxia limits cellular activity, and alterations in immune function may reduce resistance to infection [11,13,19]. As a result, even technically atraumatic extraction may become biologically hazardous when performed within a severely compromised irradiated field.

Radiation also directly affects bone-forming and bone-remodeling cells. Experimental studies show that radiation impairs osteoblast viability, proliferation, and differentiation, reducing the capacity for new bone formation [18]. In parallel, radiation-induced alterations in osteoclast function may disrupt the normal balance of bone remodeling and repair [20]. These changes contribute to reduced bone turnover and diminished regenerative potential within irradiated mandibular bone. Consequently, the extraction socket may be less able to undergo the coordinated remodeling required for normal healing, particularly in regions exposed to high radiation doses.

A critical distinction must therefore be drawn between the resolution of acute radiation toxicity and the restoration of long-term healing capacity. While acute mucositis, edema, and inflammation often resolve within weeks to months after completion of RT, normalization of the oral mucosal surface should not be conflated with recovery of the underlying bone and periosteal microenvironment. Clinically intact mucosa may mask persistent hypovascularity, hypocellularity, fibrosis, and metabolic compromise in deeper tissues. Thus, a favorable clinical appearance does not reliably indicate restored wound-healing potential.

This distinction is particularly important in the context of extraction timing. In the immediate post-RT period, surgical intervention may be complicated by acute mucosal toxicity, pain, edema, and inflammation. Chronic fibrovascular and fibrotic alterations may subsequently persist or evolve, but their temporal course has not been translated into a validated time-dependent ORNJ risk trajectory. Accordingly, resolution of acute toxicity should not be interpreted as complete biological recovery, nor should increasing elapsed time be assumed to confer either increasing protection or increasing risk. From a radiobiological standpoint, the relevant question is how acute and chronic components of radiation injury evolve and interact with local radiation exposure, tissue condition, and surgical trauma over time.

**Figure 1:**
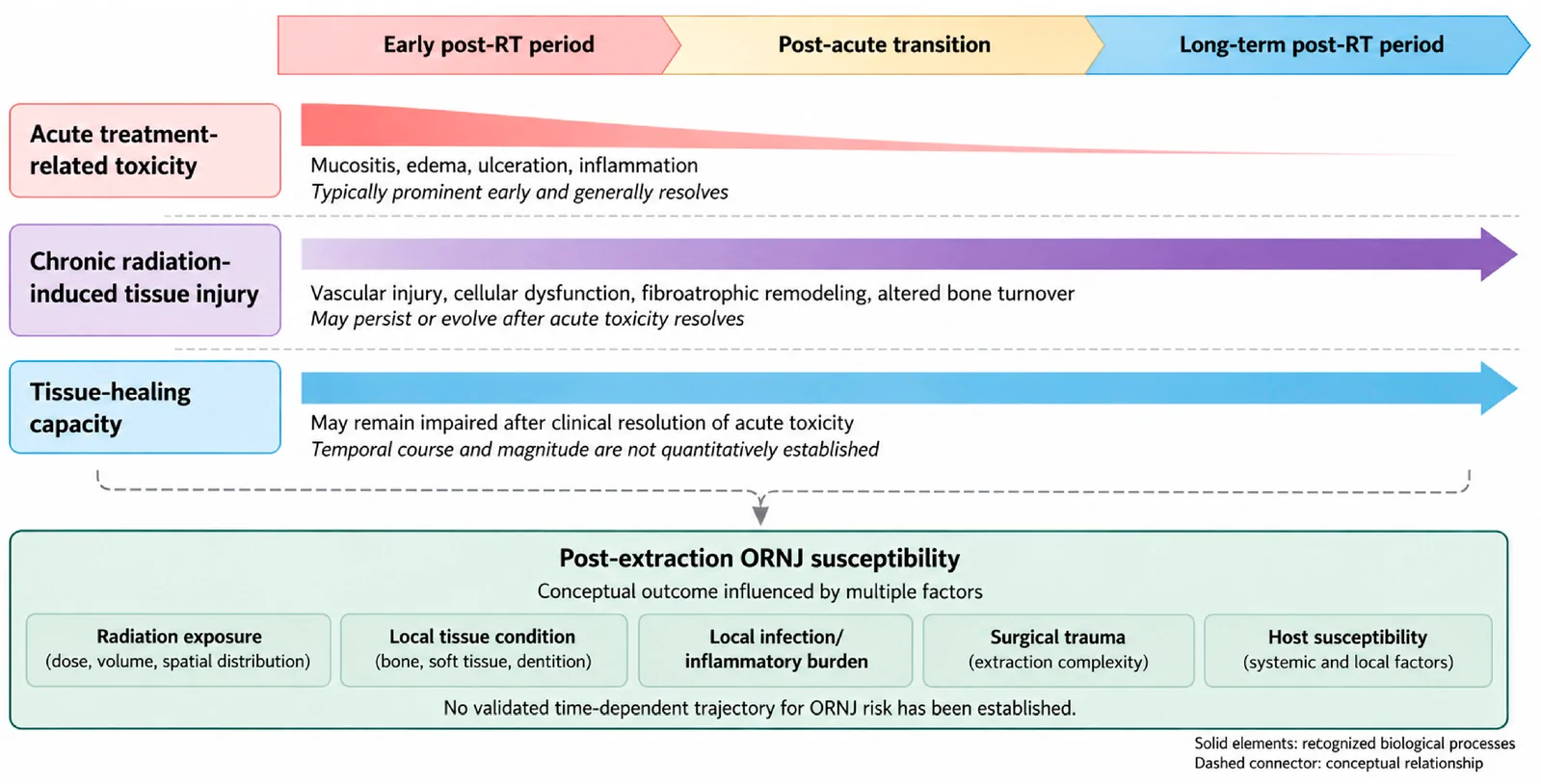
Conceptual framework of temporal components of radiation injury relevant to post-radiotherapy dental extraction. Acute treatment-related toxicity is typically most prominent during the early post-RT period and generally resolves, whereas vascular, cellular, fibroatrophic, and bone-remodeling abnormalities may persist or evolve after acute toxicity has subsided. These biological processes may contribute to sustained impairment of tissue-healing capacity, but their magnitude and temporal course vary among patients and tissues. ORNJ susceptibility is therefore not depicted as a quantitative or monotonic time-dependent trajectory. Rather, post-extraction risk is conceptualized as arising from the interaction of radiation exposure, local tissue condition, infection, surgical trauma, and host susceptibility. The figure is schematic and hypothesis-generating; it does not represent longitudinal measurements, validated temporal risk functions, or clinical decision-making thresholds. Abbreviations: ORNJ, osteoradionecrosis of the jaws; RT, radiotherapy. Note: The temporal categories are schematic and do not represent validated biological phases, ORNJ risk thresholds, or clinical decision-making cutoffs.

The mandible is particularly vulnerable in this regard because of its dense cortical structure, relatively limited collateral blood supply, and frequent exposure to clinically significant radiation doses during treatment of oral cavity, oropharyngeal, nasopharyngeal, and other head and neck malignancies [4,5,14,15]. Modern dose–volume analyses consistently demonstrate strong associations between mandibular radiation exposure and subsequent ORNJ development [4,5,14,15]. These observations suggest that mandibular radiation exposure is not merely a technical treatment characteristic but an important determinant of the biological environment relevant to residual healing capacity. Figure 1 provides a conceptual framework for the temporal components of radiation injury relevant to post-RT dental extraction and distinguishes established biological processes from hypothesized implications for tissue-healing capacity and ORNJ susceptibility.

Contemporary radiation biology supports a model of persistent radiation-induced tissue injury characterized by vascular compromise, impaired cellular function, fibrosis, and altered bone remodeling [11,13,18,19,20], which may contribute to dose-dependent biological vulnerability. These observations argue against assuming that elapsed time alone restores the healing capacity of irradiated jawbone, while also not implying that early post-RT extraction is inherently safer. They provide the basis for asking whether the period after resolution of acute toxicity may differ biologically from later phases of radiation injury, without presuming that such differences translate into a clinically safer extraction interval.

## 5. Could the Early Post-Acute Period Represent a Biologically Distinct Phase?

If irradiated tissues do not predictably regain healing capacity over time, an important question arises: might the period after resolution of acute radiation toxicity nevertheless represent a biologically distinct phase of post-RT tissue injury? This question is clinically relevant because extraction timing remains one of the few potentially modifiable factors in the management of irradiated patients. During the immediate post-RT period, active mucositis, edema, ulceration, pain, and soft-tissue injury may impair surgical closure and wound healing [1,7,8,9]. Accordingly, extractions are generally deferred during periods of active toxicity unless infection, uncontrolled symptoms, or another compelling indication necessitates intervention [1,9].

Avoiding extraction during active acute toxicity should not, however, be conflated with evidence supporting a prolonged waiting interval. After acute mucosal reactions resolve, the biological state of irradiated tissues may continue to evolve. Contemporary fibroatrophic models describe persistent endothelial dysfunction, microvascular alterations, extracellular-matrix remodeling, and fibrosis long after the acute phase has subsided [13,19]. These mechanisms raise the possibility that the early post-acute period represents a biologically distinct transition phase, in which acute epithelial and inflammatory injury has clinically subsided while chronic fibroatrophic remodeling continues to evolve. This possibility is mechanistically plausible, but it has not been demonstrated to constitute a clinically advantageous extraction window. In particular, radiobiological models of progressive late injury cannot by themselves establish that extraction performed during this interval carries a lower ORNJ risk than extraction performed later. The hypothesis therefore concerns temporal differences in tissue biology and the possibility that the clinical implications of extraction timing may vary across phases of radiation injury [7,8,21], rather than evidence of a preferred clinical extraction interval.

Current evidence does not delineate a universally safe or preferential window for early dental extraction following RT. Systematic reviews consistently underscore substantial heterogeneity in extraction timing, patient selection, radiation exposure, surgical protocols, and outcome ascertainment, precluding the determination of an optimal post-RT extraction interval [7,8]. In a prospective pilot study, Ward et al. evaluated 50 patients, 30 of whom underwent planned dental extractions at a median of 64.5 days (range, 13–152 days) after RT completion [21]. The two-year cumulative incidence of exposed bone was 40% (95% CI, 22–58%) among patients undergoing post-RT extraction, compared with 7% (95% CI, 0–22%) among those who did not undergo planned extractions. Confirmed osteoradionecrosis occurred in eight patients overall, with a two-year cumulative incidence of 23% (95% CI, 8–38%) in the extraction group. The interval between RT completion and extraction was not significantly associated with exposed bone when analyzed continuously per additional week using univariable competing-risk regression (HR, 1.01; 95% CI, 0.91–1.11). These findings should be interpreted in light of the study’s prospective pilot design and exploratory objectives. The modest number of extracted patients and exposed-bone events limited the precision of timing-related estimates, while the analysis did not specifically evaluate potentially nonlinear temporal relationships. Furthermore, patient self-selection into extraction groups, variation in radiation exposure, relatively limited follow-up, and the distinction between exposed bone and confirmed ORNJ warrant consideration. Because the study did not directly compare early with substantially delayed extraction, its findings cannot establish the relative safety of different post-RT waiting intervals. Nevertheless, this prospective investigation provides valuable clinical evidence of complications following relatively early extraction and reinforces the need for adequately powered studies evaluating extraction timing alongside radiation exposure and biological vulnerability.

Evidence likewise does not establish that progressively longer waiting confers increasing protection; systematic reviews emphasize substantial heterogeneity and do not permit definition of an optimal post-RT extraction interval [7,8]. Interpretation of timing effects is further complicated by differences in patient characteristics, radiation modalities, mandibular dose exposure, extraction indications, surgical protocols, antibiotic regimens, and ORNJ definitions [6,7,8,9].

A single retrospective cohort of patients undergoing post-CCRT dental extraction has suggested that mandibular radiation dose may modify the association between extraction timing and ORNJ risk [22]. In that study, mandibular radiation dose was the principal predictor of ORNJ, and a statistically significant dose–timing interaction was reported. ORNJ incidence remained low across extraction intervals at lower dose levels, whereas delayed extraction was associated with higher ORNJ rates in more heavily irradiated mandibular regions [22]. These observations are hypothesis-generating and require independent external validation; they should not be interpreted as establishing a reproducible dose-dependent timing effect or as evidence that earlier extraction is preferable.

The biological plausibility of this hypothesis derives from the established relationship between radiation exposure and tissue injury. Radiation dose influences the extent of endothelial injury, vascular compromise, hypoxia, fibrosis, and impaired bone remodeling [11,13,18,19,20]. Thus, an ostensibly identical extraction procedure may occur in markedly different biological environments, contingent on the radiation exposure of the mandibular bone. In lower-dose regions, residual reparative capacity may be sufficient to support healing across a range of extraction intervals, whereas greater radiation-induced tissue injury may alter the biological consequences of surgical trauma, providing a plausible basis for investigating whether the relevance of extraction timing differs across levels of radiation exposure [4,5,14,15,22].

From a clinical and interpretive standpoint, these findings caution against reliance on fixed temporal thresholds. The relevance of extraction timing should instead be considered in conjunction with mandibular radiation exposure, local tissue status, extraction indication, surgical trauma, and patient-specific factors affecting wound healing [1,7,8,9,16,22]. Current evidence establishes neither a universally safe or preferential early extraction window nor a universally protective waiting period; future studies should determine whether the consequences of extraction timing vary across patterns of radiation injury and tissue vulnerability. This framework also motivates the methodological considerations addressed in the next section, because apparent advantages of delayed extraction may, under some observational study conditions, reflect selection processes rather than biological recovery.

## 6. Potential Sources of Bias in Observational Studies of Post-Radiotherapy Extraction Timing

Interpretation of the post-RT extraction-timing literature is complicated by the non-random nature of extraction timing. Patients undergoing delayed extraction are not simply earlier-extraction patients observed at a later time point; depending on study design and clinical selection processes, they may represent a subgroup that has survived competing events and remained clinically eligible for extraction during follow-up. Such cohort evolution creates the potential for selection-related bias and complicates causal interpretation of associations between elapsed time and ORNJ risk [23,24,25,26]. Importantly, the presence and magnitude of these biases have not been directly quantified across most published extraction-timing studies. They should therefore be regarded as plausible methodological explanations that require study-specific assessment rather than as demonstrated causes of the observed timing associations.

One plausible mechanism is selection bias arising from differential eligibility for later extraction. Patients who develop severe ORNJ, advanced fibrosis, persistent mucosal breakdown, recurrent infection, local recurrence, or who die during follow-up may be less likely to remain eligible for, or to undergo, delayed extraction. Consequently, patients represented in later-extraction strata may differ systematically from those undergoing earlier intervention in characteristics related to their underlying risk profile [23,26]. Whether this mechanism materially affected the findings of any individual published study depends on its eligibility criteria, exposure definition, follow-up structure, and handling of competing events and therefore requires study-specific evaluation.

A related concern is survivor bias. Eligibility for delayed extraction necessarily requires that a patient remain alive and clinically eligible until the later time point. Patients who die, experience recurrence, develop severe treatment-related complications, or undergo earlier intervention cannot subsequently enter a delayed-extraction group. This structure can preferentially retain patients with greater clinical stability and may thereby create differences between earlier- and later-extraction cohorts that are unrelated to biological recovery [23,24]. However, the extent to which survivor bias contributed to reported timing associations has not been directly quantified in most published studies and should therefore be considered a potential source of distortion rather than an empirically established explanation.

Beyond survival itself, clinical suitability may introduce an additional selection mechanism. Decisions to undertake or defer extraction can be influenced by factors such as mucosal integrity, fibrosis, oral hygiene, nutritional status, treatment-related complications, and access to ongoing dental surveillance. If these characteristics differ across extraction-timing strata, later-extraction cohorts could become enriched for patients with more favorable clinical profiles, creating a “healthy candidate” effect [23,26]. Because such characteristics have not been consistently measured or compared across timing groups, however, this mechanism should be regarded as a plausible source of selection bias rather than an established feature of delayed-extraction cohorts.

A further conceptual analogy is depletion of susceptibles, whereby individuals at higher baseline risk experience an outcome earlier, leaving a progressively lower-risk population under observation [23,26]. Applied to post-RT extraction studies, patients with greater underlying susceptibility to ORNJ could be less likely to remain represented in later-extraction strata because of earlier complications, competing events, or prior intervention. If such differential attrition occurs, the composition of delayed-extraction cohorts may shift toward individuals with more favorable biological or clinical characteristics over time. However, depletion of susceptibles has not been directly demonstrated as a mechanism in the published extraction-timing literature and should therefore be considered a plausible source of cohort evolution rather than an established explanation for lower ORNJ rates with delayed extraction.

Another methodological limitation is that many extraction-timing studies do not systematically characterize or compare the biological features of extraction sites across timing strata. Variables such as whole-mandible dose–volume characteristics, regional or extraction-site radiation exposure, mucosal integrity, periodontal status, active infection, fibrosis, and local bone quality are incompletely reported or adjusted for in much of the available literature [4,5,14,15,16,22]. This limitation creates the potential for site-level confounding because the clinical factors influencing whether and when extraction is undertaken may also influence subsequent ORNJ risk. For example, sites selected for later elective extraction could differ from those requiring earlier intervention because of pain, infection, periodontal breakdown, or other adverse local conditions. Where these characteristics are not measured and compared, observed differences between early- and delayed-extraction groups cannot be attributed confidently to elapsed time alone. Importantly, this possibility reflects incomplete control of site-level heterogeneity rather than direct evidence that later-extraction cohorts are systematically more biologically favorable.

**Figure 2:**
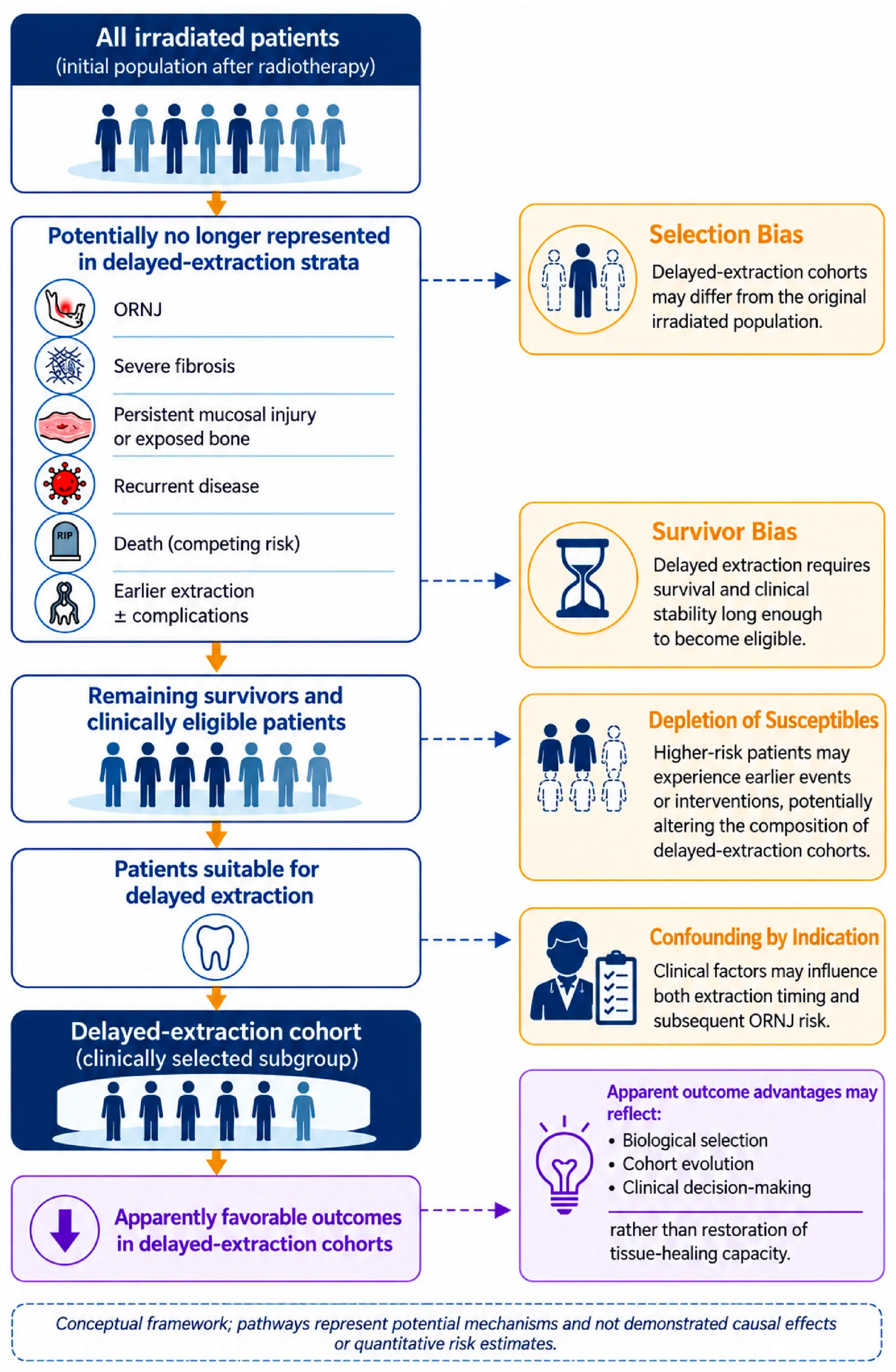
Conceptual framework illustrating how selection bias, survivor bias, depletion of susceptibles, and confounding by indication may contribute to the apparent safety of delayed post-radiotherapy dental extraction. Patients undergoing dental extraction years after radiotherapy represent a progressively selected subgroup that has survived competing events and remained clinically suitable for intervention. Individuals with severe radiation-related complications, recurrence, death, or other adverse outcomes may be removed from the population eligible for delayed extraction over time. Consequently, apparently favorable outcomes observed among delayed-extraction cohorts may partly reflect biological selection, cohort evolution, and clinical decision-making rather than restoration of irradiated tissue-healing capacity. Adapted from concepts of selection bias, survivor bias, depletion of susceptibles, confounding by indication, and causal-inference methodology [23,24,25,26]. Abbreviations: ORNJ, osteoradionecrosis of the jaws. Note: This figure presents a conceptual framework based on established epidemiological principles. The pathways shown represent potential mechanisms by which observational study design and clinical selection could influence associations between extraction timing and ORNJ risk; their presence and magnitude have not been established across the reviewed extraction-timing studies. The figure does not represent quantitative risk estimates or proven causal relationships.

Figure 2 conceptually illustrates the potential influence of selection bias, survivor bias, depletion of susceptibles, and confounding by indication on delayed-extraction cohorts.

Clinical decision-making introduces further potential for confounding. Extraction timing is rarely determined at random; instead, it reflects clinical judgment informed by factors such as fibrosis, mucosal integrity, exposed bone, infection, trismus, oral hygiene, and surgical accessibility. Importantly, the clinical indication for extraction is itself dynamic: dental disease, infection, pain, periodontal breakdown, non-restorability, and radiation-related tissue changes may emerge or progress during post-RT follow-up and may influence both when extraction is undertaken and subsequent ORNJ risk. The indication should therefore be considered a potentially time-varying determinant of treatment selection rather than a fixed baseline characteristic. Failure to characterize its onset, evolution, and severity may produce confounding by indication when extraction-timing groups are compared [23,26]. However, the extent to which such confounding influenced individual published timing studies cannot be established where the relevant determinants of treatment selection were not reported or formally evaluated.

These potential sources of bias are particularly relevant when extraction timing is analyzed in isolation. Variables such as radiation dose, irradiated mandibular volume, local tissue condition, surgical technique, systemic comorbidity, smoking status, nutritional status, and overall oral health may influence both extraction timing and ORNJ risk, while the indication for extraction may itself emerge or change during follow-up [6,7,8,9,16,22]. Inadequate characterization of these baseline and time-varying factors may lead observed differences in ORNJ incidence to be attributed to elapsed time when they are partly or wholly explained by the biological and clinical processes that determine whether and when extraction occurs [23,26].

The conceptual implications are substantial. If delayed-extraction cohorts become progressively enriched with biologically or clinically favorable survivors and extraction sites, an observed association between delayed extraction and lower ORNJ incidence could arise partly from cohort composition rather than from restoration of tissue-healing capacity. We use the term “waiting-period illusion” to describe this hypothetical interpretive scenario, not to imply that such bias has been demonstrated across the existing literature. Under this framework, elapsed time may function partly as a marker of patient and site selection rather than as a direct biological determinant of healing [23,26]. The presence and magnitude of any such effect should therefore be evaluated empirically within individual studies.

These considerations may also be relevant when interpreting the hypothesis that the association between extraction timing and ORNJ risk varies according to radiation exposure [22]. If the biological and clinical characteristics of patients or extraction sites differ across timing strata, changes in cohort composition over follow-up could influence the observed relationship between elapsed time and ORNJ risk. Such selection processes therefore represent one possible explanation for heterogeneity in timing associations, but their contribution to the dose–timing interaction reported in [22] was not directly evaluated and should not be inferred from that study.

Another potential concern is immortal time bias, which can arise when extraction timing is classified using a future event or a predefined post-RT interval while exposure status is treated as fixed from baseline. Under such designs, patients categorized as undergoing delayed extraction must remain alive and clinically eligible until the relevant interval or extraction event is reached. Events occurring before that time may therefore be handled differently across exposure groups, potentially favoring the delayed-extraction category if time-dependent exposure status is not modeled appropriately [24,25]. Whether immortal time bias is actually present depends on the study’s definition of time zero, exposure classification, risk-set construction, and analytic approach and must therefore be assessed on a study-specific basis.

Importantly, these considerations do not show that delayed extraction is ineffective, nor do they establish the superiority of earlier intervention. Rather, they highlight the limitations of conventional observational analyses and caution against causal interpretation of timing effects. A central methodological challenge is that extraction timing is inherently non-random, emerging from complex interactions among tissue biology, clinical judgment, patient characteristics, disease trajectory, and treatment-related factors [23,26].

Consequently, future investigations should move beyond simple comparisons of early versus delayed extraction. Studies should incorporate mandibular dose–volume parameters, detailed local tissue assessments, extraction indications, competing-risk methodologies, and time-dependent analytical approaches that permit evaluation of potential dynamic patient and site selection [24,25,26]. Where feasible, causal-inference frameworks designed to emulate target trials may help distinguish true timing effects from associations distorted by cohort evolution, treatment selection, or other time-dependent sources of bias [25,26].

Taken together, these considerations indicate that observed associations between extraction timing and ORNJ risk may reflect both biological effects and methodological processes. Survivor selection, clinical decision-making, immortal time, cohort evolution, and site-level biological non-comparability may each distort timing associations under the relevant study conditions, but their presence and magnitude must be established within individual studies rather than be assumed across the literature. Recognizing these potential sources of bias is therefore important for interpreting existing observational findings and for designing future studies capable of distinguishing genuine temporal effects from artifacts of cohort construction and treatment selection.

## 7. Dose-Dependent Biological Vulnerability: Moving Beyond Time-Centric Models

The preceding sections suggest that elapsed time following RT should not be considered in isolation when interpreting post-extraction ORNJ risk. Contemporary radiation biology indicates that irradiated tissues may sustain chronic vascular, cellular, and fibroatrophic alterations well beyond treatment completion [11,13,18,19,20]. At the same time, observational associations between delayed extraction and ORNJ risk may be susceptible to survivor selection, depletion of susceptibles, confounding by indication, and other forms of cohort evolution when the relevant study conditions are present [23,24,25,26]. These mechanisms have not been demonstrated uniformly across the extraction-timing literature and should therefore be regarded as potential sources of distortion rather than established explanations for favorable outcomes in delayed-extraction cohorts. Together, these biological and methodological considerations support interpreting elapsed time within a broader framework of dose-dependent biological vulnerability rather than as a stand-alone determinant of post-RT extraction risk.

Among the factors considered in this review, radiation exposure has one of the most consistently reported associations with ORNJ. Clinical and dosimetric studies have reported associations between mandibular radiation exposure and subsequent ORNJ development [5,6,14,15]. This relationship is biologically plausible, as radiation dose directly governs the extent of endothelial injury, microvascular compromise, hypoxia, extracellular-matrix remodeling, fibrosis, and impairment of bone turnover [11,13,18,19,20]. Thus, radiation dose is not merely a treatment parameter; it fundamentally shapes the biological environment for post-extraction wound healing.

Mandibular radiation exposure cannot be adequately represented by a single interchangeable dosimetric parameter. Published studies have evaluated whole-mandible mean dose, maximum dose, dose–volume metrics, and measures intended to characterize exposure of the clinically involved region [4,5,14,15,16]. These parameters capture different aspects of radiation exposure: mean dose summarizes the average dose burden but obscures spatial heterogeneity; maximum dose reflects peak exposure without characterizing the volume of irradiated bone; and dose–volume parameters quantify the volume receiving specified dose levels but do not identify where that exposure occurs within the mandible. For extraction-related ORNJ, regional or extraction-site dose is conceptually more anatomically aligned with the site of surgical injury, although direct clinical validation of socket-level dosimetry remains limited. These metrics should therefore be regarded as non-equivalent, and no single mandibular dose parameter or numerical threshold is universally validated for predicting post-extraction ORNJ. Where treatment-plan data are available, interpretation should consider regional or extraction-site exposure, when it can be reliably determined, together with whole-mandible dose–volume information [4,5,14,15,16,22].

Against this broader dose–ORNJ evidence, the proposed dose–timing interaction currently rests on a single retrospective cohort of patients undergoing post-CCRT dental extraction [22]. That study reported a statistically significant interaction between mandibular radiation dose and extraction timing, with mandibular radiation dose identified as the dominant predictor of ORNJ. At lower mandibular mean-dose levels, ORNJ incidence remained low regardless of extraction timing; in contrast, delayed extraction was associated with substantially higher ORNJ rates at higher mandibular mean-dose levels [22]. These findings are hypothesis-generating, require external validation, and should not be construed as definitive evidence supporting early extraction; rather, they raise the possibility that the clinical significance of extraction timing may depend on the degree of underlying biological vulnerability.

This interpretation may account for inconsistencies observed in the literature. If radiation dose is the primary determinant of tissue injury, then the effect of extraction timing is likely to vary across different biological environments. In mandibular segments exposed to relatively modest radiation doses, tissue may retain sufficient reparative capacity to permit successful healing across a range of extraction intervals. Conversely, identical procedures performed in heavily irradiated segments may encounter a profoundly compromised environment—marked by persistent hypoxia, fibrosis, impaired bone remodeling, and diminished regenerative reserve—where timing may play a more pronounced role in determining risk.

Accordingly, post-RT extraction timing should not be considered an isolated determinant of ORNJ risk. Rather, timing appears to operate within a multidimensional framework shaped by radiation dose, irradiated volume, local tissue condition, extraction indication, surgical trauma, infection burden, and host-related factors, including systemic determinants of tissue oxygenation and healing capacity [22,27,28]. This perspective shifts the clinical question from pursuing a universally safe interval to assessing the specific biological circumstances in which extraction is performed. Thus, two extractions undertaken at identical post-RT intervals may entail markedly different risks, contingent on the underlying characteristics of the irradiated mandibular site.

This paradigm shift has important conceptual and methodological implications. The relevant question is no longer whether a universal waiting period can be identified, but whether extraction timing has different meanings across distinct biological contexts. A tooth extracted one year after RT from a mandibular region with relatively limited radiation exposure is not biologically equivalent to one extracted at the same interval from a region with greater radiation exposure, particularly when accompanied by fibrosis or infection [4,5,14,15,22]. Accordingly, extraction timing should be interpreted as a conditional risk modifier rather than a stand-alone protective or harmful exposure. Future studies should therefore evaluate timing within frameworks that incorporate site-specific dosimetry, local tissue status, extraction indication, competing risks, time-dependent exposure structure, and causal-inference principles [24,25,26,27].

These observations support a vulnerability-based interpretation in which the relevance of extraction timing depends on the biological and dosimetric context in which the procedure is undertaken [1,4,5,6,7,8,9,14,15,16,22,28]. This perspective provides the conceptual basis for the risk-adapted framework developed in the following section.

## 8. From Critique to Practice: A Risk-Adapted Framework for Future Research and Clinical Decision-Making

The preceding sections do not imply that extraction timing is clinically irrelevant; rather, they indicate that timing should not be interpreted in isolation. A practical framework for post-RT dental extraction should therefore move from fixed waiting periods toward individualized, risk-adapted decision-making. In this model, elapsed time after RT is considered alongside the available mandibular dose distribution, local tissue condition, dental indication, surgical complexity, and host-related healing capacity. Practically, this framework can be operationalized across three interrelated domains: dosimetric exposure, characterized by the available mandibular dose distribution, including whole-mandible dose–volume information and, when reliably determined, regional or extraction-site exposure; local and systemic modifiers of healing, including mucosal status, infection, fibrosis, smoking, diabetes, anemia, nutrition, and follow-up adherence; and procedure-related trauma, determined by extraction complexity, surgical technique, extent of periosteal stripping, and feasibility of tension-free closure.

Crucially, this framework is intended to translate the preceding biological and epidemiological considerations into structured clinical risk assessment rather than establish a new timing rule. Elapsed time should therefore be interpreted alongside the dosimetric, site-specific, host-related, and procedural determinants described above, without implying that any single timing threshold or dosimetric parameter is independently validated for clinical decision-making.

Clinically, prevention remains the most reliable strategy. Comprehensive dental evaluation before RT, treatment of restorable disease, removal of teeth with poor long-term prognosis when appropriate, and structured lifelong dental surveillance remain central to reducing the need for high-risk post-RT extraction [1,7,8,9,27]. When extraction after RT is unavoidable, the available radiation dose distribution should be reviewed whenever possible, with consideration of whole-mandible dose–volume information and, when reliably ascertainable, exposure of the involved mandibular region. Greater radiation exposure should be interpreted as one component of the overall biological and clinical risk context rather than through a universally applicable high-versus-low dose threshold [4,5,14,15,22].

A risk-adapted clinical approach should also include systematic evaluation of the extraction site. Active infection, mucosal ulceration, exposed bone, severe fibrosis, trismus, periodontal instability, or poor oral hygiene may indicate a hostile wound-healing environment. Conversely, clinically intact mucosa, controlled infection, favorable access, and the possibility of primary tension-free closure may support a more favorable surgical context. Patient-level factors such as smoking, diabetes, anemia, malnutrition, immunosuppression, and adherence to dental follow-up should be proactively addressed to optimize surgical outcomes whenever feasible, as emerging evidence suggests that host-related biological factors, including low pre-treatment hemoglobin levels, may substantially influence ORNJ susceptibility [1,6,7,8,9,16,28,29]. Consistent with this multidimensional approach, recent international multidisciplinary consensus emphasizes comprehensive oral health assessment, patient education, and coordinated care throughout the head and neck cancer treatment pathway [30].

In this framework, timing becomes one component of a broader biological assessment. Extraction during active acute mucosal toxicity should be avoided unless urgent infection or uncontrolled symptoms require immediate intervention [1,9]. However, prolonged delay should not be assumed protective solely on temporal grounds, particularly when the affected tooth remains a persistent source of pain or infection and the available dose distribution indicates substantial exposure of the involved mandibular region. Rather than applying a universal waiting interval, clinicians should balance resolution of acute toxicity against the ongoing dental indication and local tissue status while considering the available mandibular dose distribution, including regional or extraction-site exposure when it can be reliably determined, without relying on a single unvalidated dose threshold.

When surgery is required, the procedure should be approached as high-risk dentoalveolar surgery rather than routine extraction. Conservative alternatives, including endodontic therapy, restorative treatment, periodontal stabilization, and decoronation or coronectomy-like approaches where appropriate, should be considered before extraction [1,9]. If extraction is unavoidable, atraumatic technique, smoothing of sharp bone edges, minimization of periosteal stripping, primary tension-free soft-tissue closure when feasible, and close postoperative surveillance are important procedural considerations. Evidence concerning adjunctive prophylactic interventions remains heterogeneous. A recent systematic review and meta-analysis reported lower pooled ORNJ frequencies among patients receiving antibiotics, pentoxifylline/tocopherol, or hyperbaric oxygen than among those receiving no adjunctive intervention; however, substantial methodological limitations and the scarcity of direct comparisons preclude reliable conclusions regarding their comparative effectiveness [31]. Importantly, the ISOO–MASCC–ASCO guideline distinguishes these interventions. Peri-procedural antibiotic prophylaxis is recommended for patients at elevated ORNJ risk, based on informal consensus and low-quality evidence. Pentoxifylline/tocopherol carries a weak evidence-based recommendation for selected cancer-free patients undergoing invasive dental procedures, particularly when the intervention site received ≥50 Gy, provided no contraindications exist. Conversely, routine prophylactic hyperbaric oxygen is not recommended, although it may be considered in selected patients undergoing procedures in regions where a substantial volume of jawbone received >50 Gy [1]. Recent guidance on implementation of these recommendations further emphasizes multidisciplinary coordination and integration of guideline-based prevention into clinical practice [32].

Future research should mirror this clinical complexity. Studies should no longer ask only whether early or delayed extraction is safer. Instead, they should evaluate whether and how biological and dosimetric conditions modify the association between extraction timing and ORNJ risk. Prospective multicenter registries should retain treatment-plan data sufficient to derive and compare whole-mandible mean dose, maximum dose, relevant dose–volume parameters, and, when feasible, regional or extraction-site exposure, together with extraction location, extraction indication, surgical technique, mucosal status, fibrosis, infection, smoking, diabetes, hemoglobin, nutritional status, and follow-up duration. Adopt standardized ORNJ definitions and severity grading to improve comparability across studies [1,7,8,9].

Analytically, future studies should treat extraction timing as a dynamic clinical exposure rather than a fixed baseline characteristic. Competing-risk methods, time-dependent models, and causal inference approaches, including target-trial emulation, may help reduce immortal time bias, survivor bias, and confounding by indication [24,25,26]. The appropriate time zero depends on the causal question being addressed. For analyses estimating the effect of delaying extraction after a clinical indication has arisen, the date on which that indication is established may provide a more appropriate eligibility and treatment-assignment landmark than RT completion. However, the indication itself is a post-RT event that may be influenced by evolving dental disease, infection, radiation-induced tissue changes, recurrence, and other time-varying clinical factors that also affect subsequent ORNJ risk. It should therefore not be treated as an exogenous baseline characteristic; the clinical history leading to the indication and relevant time-varying covariates should be incorporated into the design and analysis whenever feasible. Conversely, if the estimate concerns the evolution of ORNJ risk from the completion of RT, RT completion should remain the origin of follow-up, with the emergence of an extraction indication and subsequent extraction represented as time-varying clinical processes rather than redefining baseline at the indication date. These alternative time origins address different questions and should not be treated as analytically interchangeable [24,25,26]. External validation of dose–timing interaction models is particularly important because such models may ultimately support individualized risk prediction and clinically actionable decision tools [22,27].

## 9. Limitations of the Current Evidence Base

The conclusions of this review should be interpreted in light of important limitations in the current evidence base. Most studies addressing post-RT extraction timing are retrospective, single-institution analyses characterized by heterogeneity in patient populations, radiotherapy techniques, extraction protocols, follow-up duration, and ORNJ definitions. Many also lack site-specific mandibular dosimetry, standardized assessment of local tissue status, and detailed reporting of extraction indications, surgical technique, and host-related risk factors. In addition, extraction timing is frequently analyzed as a static categorical variable despite being a dynamic clinical exposure, while the indication for extraction may itself emerge or evolve during follow-up. Failure to account for these time-dependent processes may increase susceptibility to survivor bias, depletion of susceptibles, immortal time bias, and confounding by indication. Accordingly, causal interpretation of observed associations between extraction timing and ORNJ risk remains limited. Future studies should integrate standardized ORNJ definitions, site-specific dosimetry, prospective local tissue assessment, and contemporary causal-inference methods to support individualized, evidence-based extraction timing after head and neck RT.

## 10. Conclusions

Clinical caution remains appropriate when considering dental extraction after RT, but elapsed time alone should not be interpreted as evidence of restored healing capacity. Available radiobiological and clinical evidence does not establish a universally safe extraction interval or show that progressively longer delays confer additional protection. Although the early post-acute period may represent a biologically distinct phase of radiation injury, this has not been shown to confer lower ORNJ risk than later extraction. Post-extraction risk should therefore be interpreted in relation to radiation exposure, local tissue condition, infection burden, surgical trauma, and host susceptibility; under the relevant observational study conditions, apparent timing associations may also be influenced by selection and confounding. Future research should determine whether and how extraction timing modifies ORNJ risk within these biological, dosimetric, and methodological contexts rather than presuming that any fixed waiting interval is inherently protective.

## Figures and Tables

**Table 1:** Historical evolution of concepts underlying post-radiotherapy tooth extraction timing recommendations.

Era/Concept	Key References	Principal Contribution	Relevance to Extraction Timing	Principal Limitation
Marx 3H Model	Marx, 1983	Hypovascularity, hypocellularity, hypoxia	Supported extraction avoidance	Not a timing study
Multifactorial Model	Epstein et al., 1987	Radiation injury, trauma, infection interaction	Reinforced surgical caution	Not a timing study
Pre-IMRT Clinical Practice	Store and Boysen, 2000; Nabil and Samman, 2011	Observational associations between extraction and ORNJ	Encouraged delayed extraction	Limited dosimetry and retrospective designs
Systematic Review Era	Nabil and Samman, 2012; Beaumont et al., 2021; Lajolo et al., 2021	Summarized extraction-related ORNJ risk	No validated safe interval identified	Heterogeneous evidence
Contemporary IMRT Era	Caparrotti et al., 2017; Kubota et al., 2021; Corrao et al., 2023	Emphasis on dose–volume effects and individualized risk	Shift toward dose-based assessment	Lack of prospective timing studies

Abbreviations: IMRT, intensity-modulated radiotherapy; ORNJ, osteoradionecrosis of the jaws. Note: The table summarizes the evolution of biological concepts and clinical evidence underlying post-radiotherapy extraction practices. None of the cited studies established a universally accepted or prospectively validated safe waiting period.

## Data Availability

No new data were created or analyzed in this study. Data sharing is not applicable to this article.
